# Insensitive to PTH of CD8^+^ T cells regulate bone marrow mesenchymal stromal cell in aplastic anemia patients

**DOI:** 10.7150/ijms.47273

**Published:** 2020-07-02

**Authors:** Sidan Li, Maoquan Qin, Runhui Wu, Hengxing Meng, Yixuan He, Bin Wang, Xuan Zhou, Guanghua Zhu

**Affiliations:** 1 Beijing Key Laboratory of Pediatric Hematology Oncology; National Key Discipline of Pediatrics, Ministry of Education; Key Laboratory of Major Diseases in Children, Ministry of Education; Hematology Oncology Center, Beijing Children's Hospital, Capital Medical University, Beijing, China.; 2 Zhong Wei Xin Biotechnology Co., Ltd, Tianjin, China.

**Keywords:** Aplastic anemia, mesenchymal stem cells, parathyroid hormone, CD8^+^ T cells

## Abstract

Aplastic anemia (AA) is a rare disorder characterized by the suppression of bone marrow function resulting in progressive pancytopenia. The pathogenesis of AA is complex and involves an abnormal hematopoietic microenvironment, hematopoietic stem cell/progenitor cell deficiencies, and immunity disorders. However, the underlying mechanism of the disease is still not fully uncovered. In this research, we collected both donor and patient samples and found suppressed proliferation, abnormal differentiation as well as increased apoptosis of patient mesenchymal stem cells (MSCs). Considering the close relationship of parathyroid hormone (PTH) and MSCs differentiation, further studies showed that although patients maintained normal serum PTH level, their CD8^+^ T cells possessed lower PTH receptors. The insensitive to PTH of patients' CD8^+^ T cells finally lead to reduced expression of key Wnt factors. In all, bone marrow CD8^+^ T cells may play an important role in inducing MSCs adipogenesis and osteogenesis imbalancement.

## Introduction

Aplastic anemia (AA) is a kind of hematopoiesis dysfunction caused by a variety of reasons, which finally leads to hematopoiesis failure and pancytopenia. A large number of studies have shown that viral infection, chemical poisons, drugs, ionizing radiation are important factors in the pathogenesis of AA, but the underlying mechanisms is still uncovered 1, 2. The commonly accepted theory is defined as "insect, seed and soil" which indicate the network relationship of T cells, hematopoietic stem cells (HSC) and bone marrow microenvironment. At present, the main research focuses on "insect and seed". As to the two key factors, the main treatments include immunosuppressive therapy (IST) and hematopoietic stem cell transplantation (HSCT). Although IST and allogeneic-HSCT (allo-HSCT) have shown great progress in the past 20 years, some patients still have the problem of treatment failure, which cause survival drop off and become the key problem to be solved 3, 4.

As an important component of hematopoietic microenvironment, mesenchymal stem cells (MSCs) are the precursor cells of osteoblasts, which had closed relationship with HSCs 5, 6. Our previous studies on hematopoietic microenvironment have shown that destruction of microenvironment leads to HSC number reducing, as well as lower colony-forming ability, which suggested that HSCs were affected by the bone marrow microenvironment 7-9. Furthermore, previous studies have shown osteogenesis inhibition in AA patients which indicating imbalanced differentiation of MSCs 10-12.

Parathyroid hormone (PTH) is a kind of peptide hormone. It acts on osteoblasts and osteoclasts by regulating their calcium and phosphorus metabolism. Some studies have shown that T cells also express PTH receptor (PTH-1R) 13. PTH can work on CD8^+^ T cells, these T cells then producing a large number of Wnt molecules, which activate Wnt signal transduction pathway of MSCs and finally promoting the differentiation of MSC into osteoblasts 14-16. The immune system is disordered and CD8^+^ T cells are abnormally activated in AA patients 2, 17, indicating the probability that their T cells may not respond to PTH signaling effectively. However, there are few reports exploring the relationship between T cells and hematopoietic microenvironment in AA patients.

## Material and Method

### Patient and samples

Peripheral blood (PB) and bone marrow (BM) samples were obtained from 21 severe aplastic anemia (SAA) patients and 9 donors. Serum samples were centrifuged at 500 g for 10 min and stored at -80 °C for future analysis. All donors were given exhaustive examination including BM aspiration in order to exclude congenital genetic disease. All guardians have signed the informed consent form. All medical treatments have been approved by the Ethics Committee of National Center for Children's Health, Beijing Children's Hospital.

### Isolation of bone marrow MSCs

BM cells were collected from iliac crest aspirates. MSCs isolated from patients or donors were cultured in α-Minimum Essential Medium (α-MEM; Gibco BRL, Gaithersburg, MD, USA) supplemented with 10% (v/v) fetal bovine serum (Gibco BRL) and 100 U/mL penicillin/streptomycin (Gibco BRL) at 37 °C in an atmosphere of 5% CO_2_ overnight. After 48 hr, the medium with a suspension of non-adhered cells were discarded. The medium was replaced twice a week. Upon reaching 80-90% confluence, the cells were detached with trypsin-EDTA (Gibco, Gaithersburg, MD). Passage 3 of patients or donor BM MSCs were used for subsequent experiments.

### Immunophenotype analysis

Cells were trypsinized, washed, and resuspended in PBS. Approximately 1×10^5^ cells were incubated with monoclonal antibodies against CD73 (AD2), CD105 (266), CD90 (5E10), CD45 (HI30), CD34 (581), HLA-DR (G46-6) (all were from BD Pharmingen). All incubations were performed at 4 °C for 30 min. Cells incubated with IgG isotype antibodies (MOPC-21) (BD Pharmingen) were used as negative controls. Cells were analyzed by using flow cytometer (BECKMAN COULTER CytoFLEX) and data were analyzed with Kaluza Analysis Software (version 2.1).

### Cell apoptosis assay

Annexin V-FITC/propidium iodide (annxinV/PI) apoptosis detection kits (Bio-Rad Laboratories, Inc., Berkeley, California) were used to determine the cell apoptosis according to manufacturer's instructions. Flow cytometer (BECKMAN COULTER CytoFLEX) was used to detect the apoptosis and data were analyzed with Kaluza Analysis Software (version 2.1).

### CD8^+^ T cell sorting

Peripheral blood mononuclear cells (PBMCs) were isolated by density centrifugation using Ficoll-Paque (HaoYang biological manufacture Co., Tianjin, China) per manufacturer's instructions. T cells were separated from other leukocytes by magnetic selection prior to FACS using an EasySep Human CD3 Positive Selection Kit II (StemCell Technologies; Vancouver, BC) according to the manufacturer's instructions. Subsequently positive selected T cells were stained with CD8 (RPA-T8) and CD4 (SK3) antibodies (BD pharmacy). Cells were incubated on ice prior to sorting on a BD FACS Aria II for CD8^+^ CD4^-^ T cells.

### BM MSC differentiation assay

To evaluate differentiation potential, BM-MSCs (P3) were induced to differentiate into osteoblasts and adipocyte. The induction medium for osteogenesis was Iscove's Modified Dulbecco's Medium (IMDM) (Gibco, Carlsbad, CA, USA) supplemented with 10% FBS, 10 mM β-glycerophosphate (Sigma, St Louis, MO, USA), 0.1 μM dexamethasone (Sigma), and 0.2 mM ascorbic acid (Sigma) for 3 weeks. On day 21, cultures were stained for alkaline phosphatase (ALP; Sigma) activity. The induction medium for adipogenesis was IMDM supplemented with 10% FBS, 1 μM dexamethasone, 0.5 mM 3-isobutyl-1-methylxanthine (Sigma), 0.1 mM indomethacin (Sigma), and 10 μg/ml insulin (Novo Nordisk A/S, Bagsværd, Denmark) for 2 weeks. After 3 days, the culture medium was completely replaced. The medium was then changed twice weekly thereafter. On day 14, adipogenic differentiation was demonstrated by intracellular accumulation of lipid droplets stainable with oil red O (Sigma).

### Cell counting kit-8(CCK-8) assay

Passage three BM MSCs from both SAA patients and donor were seeded at a density of 3000 cells per well into 96-well plates. Cell viability was assessed using the Cell Counting Kit-8(CCK-8, Abcam). According to instruction manual, CCK8 reagent was added to each well, and then cultured for 2,4,6,8 and 10 days. OD450 (450 nm absorbance) was read. Each assay was performed in triplicate.

### Serum protein measurement

PTH concentration was quantified by the human PTH ELISA kit (Novus Biologicals, LLC) according to the manufacturers' instructions. The concentrations of PTH for each sample were calculated and expressed as average of three identically designed experiments.

### RNA isolation, reverse transcription and real-time quantitative PCR

Patients and donor CD8^+^ T cells were directly sorted into TRIzol™ LS Reagent (Thermo Fisher Scientific). Total RNA was isolated based on the protocol provided by the manufacturer and reverse transcribed into cDNA using the Superscript First-Strand Synthesis System (Invitrogen) following the manufacturer's instructions. Real-time quantitative PCR for PTH-1R, Wnt1, Wnt5a, Wnt6, Wnt10a and Wnt10b mRNA expression were performed on the ABI 7500 Sequence Detection System (Applied Biosystems, Foster, CA) in 20 ul PCR systems containing 10 ul Power SYBR1 Green PCR Master mix (Applied Biosystems), 0.5 ul of each primer pairs (100 mM), 1 ul cDNA (40 ng RNA) and 8 ul ddH_2_O at the following conditions: 15 min of an initial denaturation at 95 °C followed by 40 cycles of 15 s at 95 °C and 1 min at 60 °C. PCR primer sequences are shown in Table 1. Each experiment was repeated three times and DDCT values were calculated from differences between the targeted genes and internal standard b-actin and averaged.

### Statistical analysis

Statistical significance was determined using Prism 6.0 software (GraphPad). Unpaired T test was used to evaluate the significance of differences between two groups. All data are mean ± SEM.

## Results

### Patient characteristics and MSC phenotype

**Figure 1:**
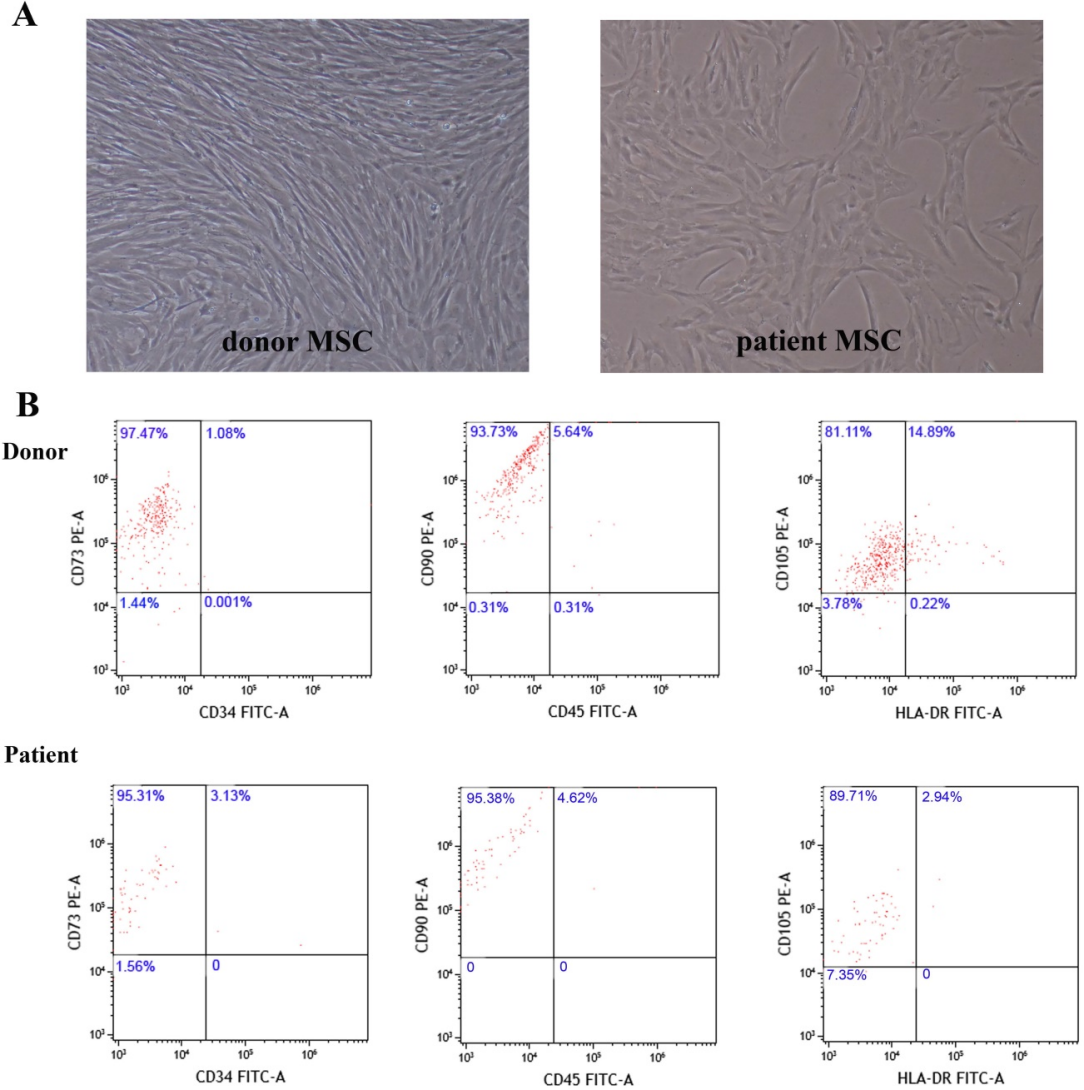
** MSCs morphology and immunophenotype of donor and patient. A.** Representative figure of donor and patient MSCs morphology. **B.** Representative dot plots showing gating strategy to identify donor and patient MSCs.

Twenty one children with SAA and nine healthy donors were enrolled in this research. The diagnosis and classification of AA were based on the International Standardized Diagnosis and Treatment Guidelines 3. All enrolled patients had negative chromosome breakage test results, and none had any characteristic of dyskeratosis congenita. Patients with clonal evolution were excluded from this study. All BM aspirates were collected after diagnosis before concomitant medication. Among these SAA patients, 11 were male, and 10 were female. They were aged at 6 years 5 months to 14 years 9 months with median age of 10 years 3 months. The period from diagnosis to HSCT was 1 to 5 months with median of 3 months. As to donors (siblings of six selective patients), four were male and five were female, aged from eight to sixteen with median age of thirteen. We compared patients and donor MSCs characteristic by using inverted phase contrast microscope and flow cytometry. Results showed that the two groups were similar in morphology and immunophenotype (Figure 1).

### Decreased proliferation ability while increased apoptosis of SAA patients MSCs

**Figure 2:**
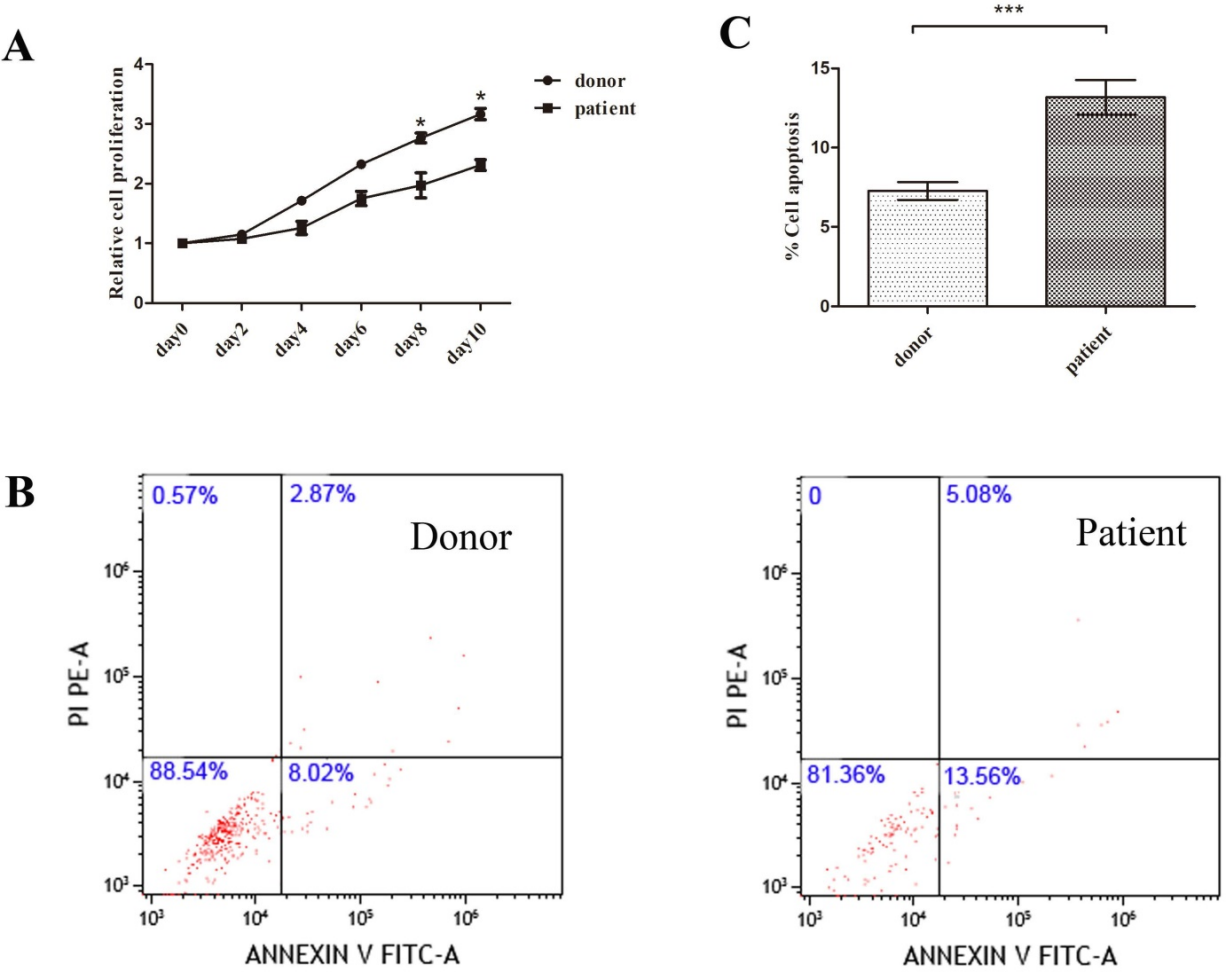
** Disadvantage of proliferation and venerable to apoptosis of patient MSCs compared to donor. A.** Proliferation of MSCs was analyzed by CCK8 assay. Data were normalized to day0 in order to eliminate starting cell number discrepancy. (N=4 for each group). **B.** Annexin V-FITC and PI labeling in donor and patient MSCs were measured by flow cytometer. **C.** Quantification of cell apoptosis rate by flow cytometry. (N=6 for each group). Data represent the mean ± SEM. Statistical significance determined using an unpaired t-test. *P<0.05, **P < 0.01, ***P < 0.001.

MSCs from patients or donors were passaged in vitro for 3 generations. In order to find out the proliferation ability of patients MSCs, we performed CCK8 assay. Although SAA patients obtained similar morphology and immunophenotype with donor, patients MSCs showed a clear disadvantage of proliferation ability compared to donor MSCs (Figure 2A).

Next we performed apoptosis assay to explore whether the decrease of patients MSCs proliferation correlated with MSCs apoptosis. The percentage of apoptosis was determined by using double-staining of death markers (AnnexinV/PI) with flow cytometry. Patients MSCs showed higher apoptosis rate compared to donor group (Figure 2B, C).

### SAA patients MSCs priority to differentiation into adipose

**Figure 3:**
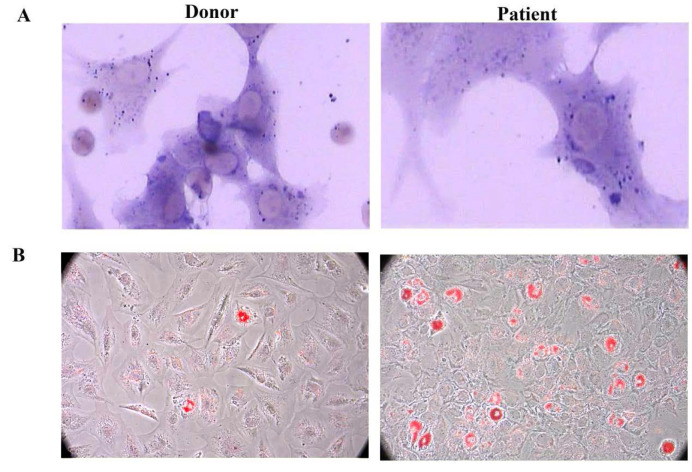
** SAA patients MSCs showed adipogenesis skewing. A.** Representative figure of donor and patient MSCs ALP staining. **B.** Representative figure of donor and patient Oil Red O staining.

After induction with different conditional media, BM-MSCs could differentiate into osteoblasts and adipocytes as detected by positive staining of ALP and Oil Red O, respectively. Compared to healthy donors, MSCs derived from patients with AA showed a propensity to differentiate into adipogenic (Figure 3).

### Patients keep normal serum PTH level while low expression of PTH-1R mRNA

**Figure 4:**
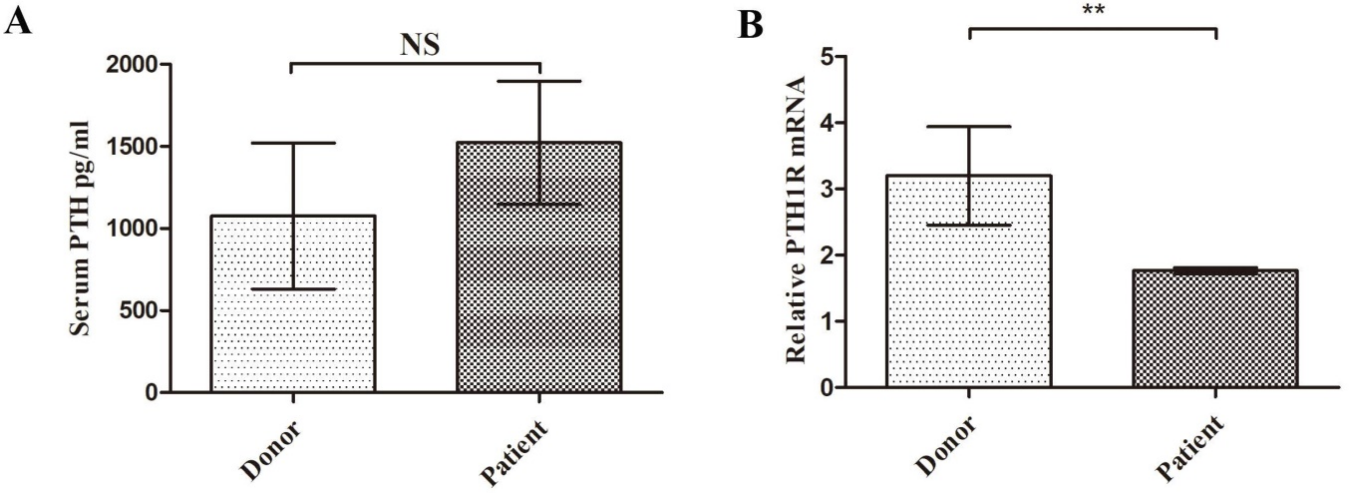
** SAA patients CD8^+^ T cells were not sensitive to PTH. A.** Serum PTH concentrations of donor and patient. (N=9 to 21 for each group) **B.** Expression of PTH-1R gene relative to GAPDH from sorted CD8^+^ T cells RNA. (N=5 to 14 for each group). Data represent the mean ± SEM. Statistical significance determined using an unpaired t-test. *P<0.05, **P < 0.01, ***P < 0.001.

Considering the important role of PTH on MSCs, we compared patient's serum PTH level with donor. Although patients MSCs showed clear disadvantage of proliferation and increased apoptosis, the serum PTH concentration appeared no significant difference between these two groups (1523.6±37.5 pg/ml vs 1075.8±44.5 pg/ml, P=0.49, Figure 4A). In order to uncover the role of T cell in SAA patients, we sorted patients or donor PB CD8^+^ T cell. Interestingly, the specific receptor of PTH, PTH-1R mRNA expression in PB T cell of SAA patients was significantly lower than donor group (Figure 4B). These findings indicated even the level of PTH was normal in patients, their T cells may not sensitive to PTH.

### T cell key Wnt factor mRNA expression

**Figure 5:**
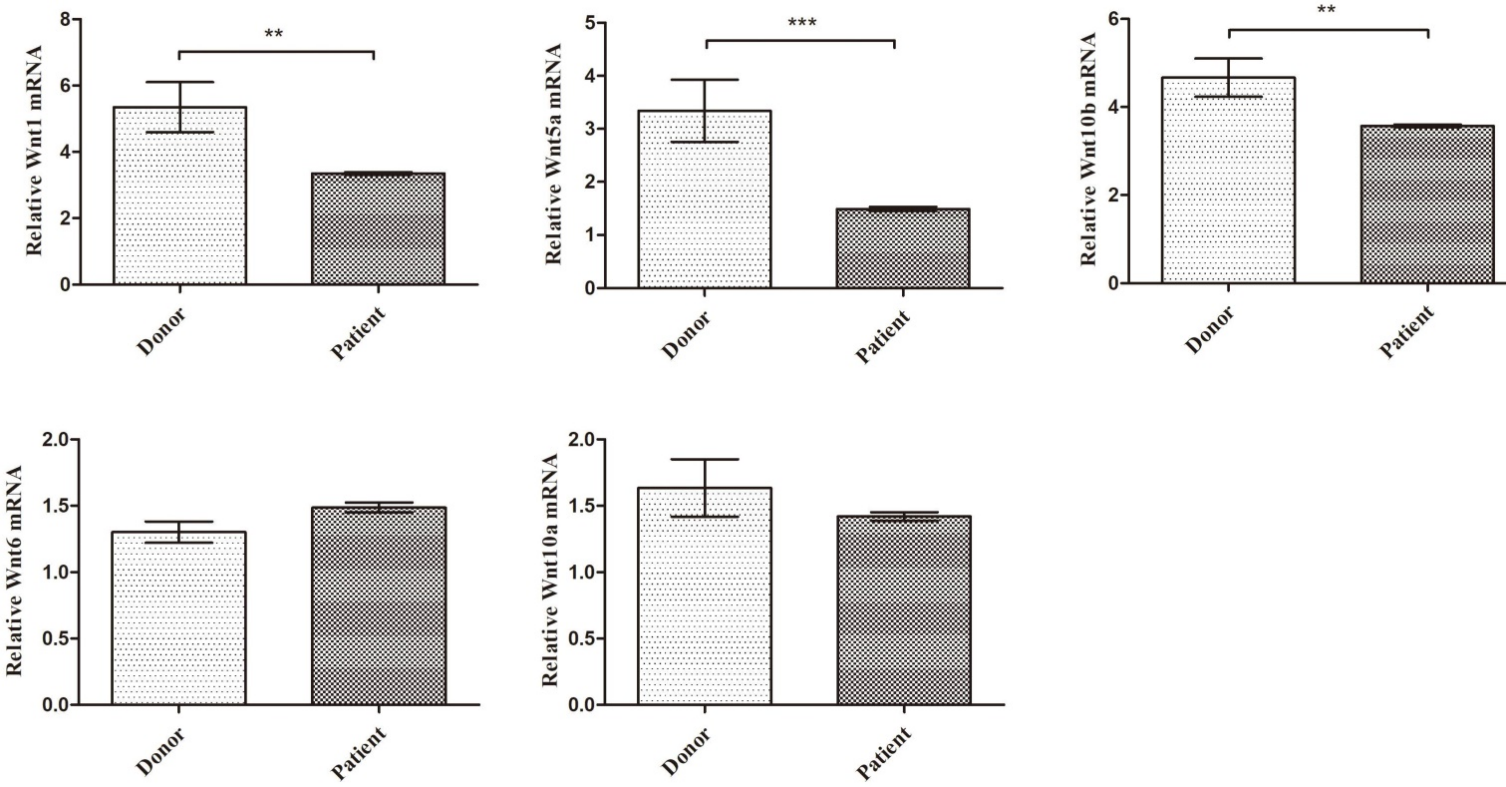
** Key Wnt factors from CD8^+^ T cells for MSC osteogenic differentiation were decreased in SAA patients.** Expression of the indicated gene-gene relative to GAPDH from sorted CD8^+^ T cells RNA. (N=8 to 14 for each group). Data represent the mean ± SEM. Statistical significance determined using an unpaired t-test. *P<0.05, **P < 0.01, ***P < 0.001.

CD8^+^ T cell is the major source of Wnt factors, which plays a key role in MSCs differentiation and proliferation. The activation of PTH and PTH-1R signaling prompts CD8^+^ T cells secreting amount of Wnt factors. Based on the above findings, SAA patients' T cells showed lower expression of PTH-1R, their Wnt protein expression may abnormal. In order to further exploration, we detected five key Wnt factor mRNA expression level. As expected, in PB CD8^+^ T cells, Wnt1, Wnt5a and Wnt10b mRNA expression were much lower than donors' (Figure 5). Collectively, these data suggest that key Wnt factors for MSCs osteogenic differentiation were decreased in SAA patients.

## Discussion

MSCs are bone marrow niche cells with self-replicating ability and multi-directional differentiation potential. As common progenitor cells of osteoblasts and adipocytes, MSCs are delicately balanced for their differentiation under normal circumstances 18, 19. Previous research showed that in AA patients, MSCs displayed an obvious imbalance between adipogenesis and osteogenesis, suggesting enhancement of adipogenesis and inhibition of osteogenesis. Hamzic cultured patients' MSCs with normal donors' CD34^+^ cells in vitro, and found that the proliferation ability of normal HSCs was significantly reduced 5. In another research, scientists found adiponectin and FABP4, which closely related to adipogenesis, were significantly increased in the mRNA level and protein level in AA patients compared with the normal controls. Adipocytes have a negative regulatory effect on HSCs in the bone marrow microenvironment, which inhibite the proliferation of HSCs in AA patients 20, 21. Compared with normal donors, MSCs of AA patients were more likely to differentiate into adipocytes and less likely to differentiate into osteoblasts 22. At the same time, Park et al. found that the expression of osteonectin, osteopontin and osteocalcin secreted by osteoblasts were significantly lower than that of normal controls, suggesting decreased osteoblasts activity 23. In our study, by using SAA patients and age-matched donor samples, we found that the proliferation of bone marrow MSCs in AA patients was significantly lower than in donors, even though their morphology and immunophenotype were similar. Moreover, compared with normal donors, the osteogenic ability of MSCs was decreased and they are prone to lipogenesis in patients which consistent with previous studies.

However, only a few researches have focus on the underlying mechanism of MSC osteogenesis inhibition until now. PTH is a peptide hormone, both MSCs and osteoblasts expressing PTH-1R exerting as the target cells of PTH 14. Some studies have shown that PTH can increase the number of nestin positive MSCs in the bone marrow and promote the differentiation of MSC into osteoblasts by enhancing Wnt and BMP signaling pathways 14. Our previous work also suggested that intermittent administration of PTH (iPTH) could rescue bone marrow niche osteoblast cells; indicating the increase of osteogenesis genes, bone trabeculae and osteoblasts activity. At the same time, iPTH induced more robust HSCs mobilization as well as enhanced HSCs implantation 7, 8. CD8^+^ T cells also express PTH receptor PTH-1R 13. PTH could directly act on CD8^+^ T cells, promoting the production of Wnt factors by T cells and finally activate the Wnt signaling pathway of MSCs. The activation of Wnt pathway drives MSC to differentiate into osteoblasts 13, 15, 16. Considering the disordered immune system and abnormal activated CD8^+^ T cells in AA patients 2, 17, we detected the serum and bone marrow samples from both patients and donors. The results showed that patients maintained normal serum PTH level while low expression of PTH-1R mRNA, suggesting that the PTH-PTH-1R pathway might down regulated in patient's T cells. Further experiment displayed that the mRNA levels of T cell key Wnt factors Wnt1, Wnt5a and Wnt10b were significantly lower than donors. The down-regulation of key Wnt factors in CD8^+^ T cells may finally lead to the lipogenesis skewing of MSCs in AA patients. However, our study still has deficiencies such as a relatively small sample size and needs to be verified by multicenter research with a more persuasive large sample size.

**Figure 6:**
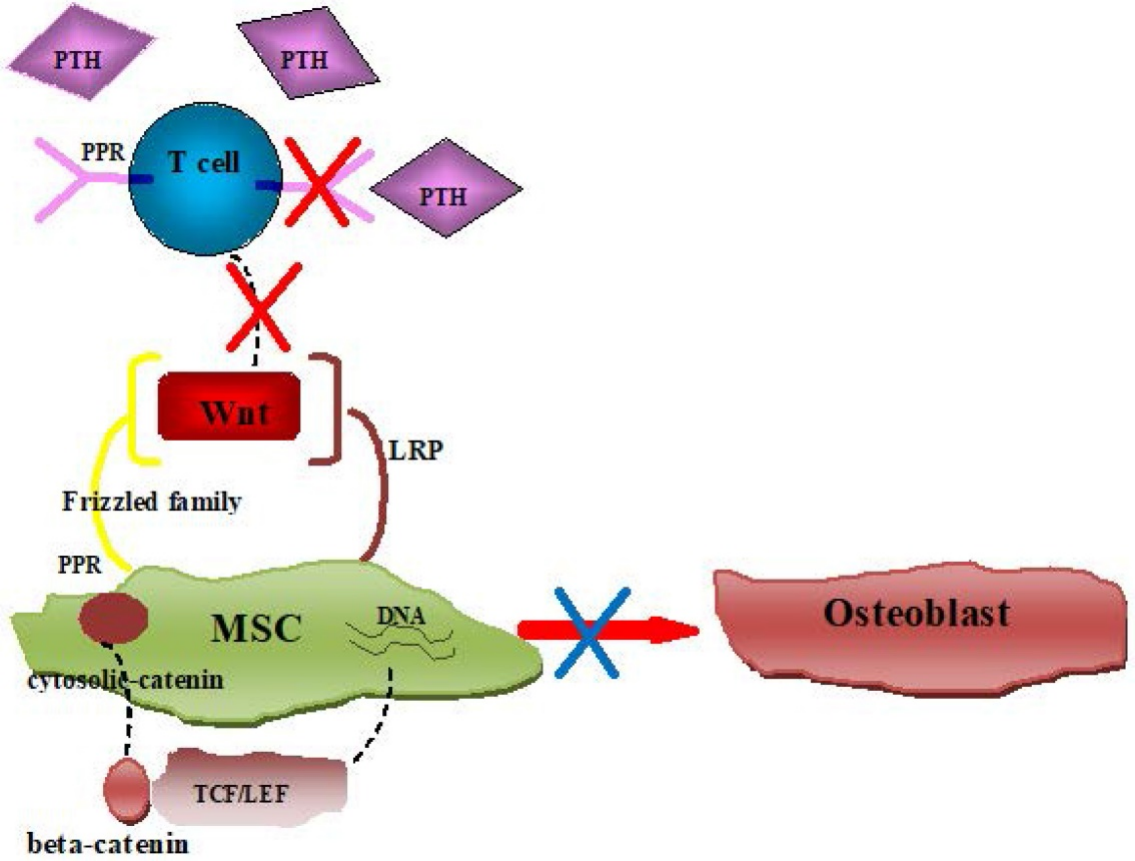
Schematic diagram showing insensitive of PTH in SAA patients' CD8^+^ T cells induced MSCs imbalance differentiation.

In all, consistent with previous data, MSC from SAA patient showed an imbalance of osteogenic and adipogenic differentiation in our research. Further exploration of the microenvironment indicated that CD8^+^ T cells were not sensitive to PTH in SAA patients due to lower expression of PTH receptor, which led to the reduction of Wnt factors. The reduction of key Wnt factors finally induced the priority of MSCs adipogenic differentiation (Figure 6). Uncover the underlying mechanism of MSCs imbalanced differentiation may provide further treatment target for SAA.

## Figures and Tables

**Table 1:** PCR primer sequences

Name	Sequence 5'-3'
GAPDH forward	CCACTCCTCCACCTTTGAC
GAPDH reverse	ACCCTGTTGCTGTAGCCA
Wnt1 forward	CGATGGTGGGGTATTGTGAAC
Wnt1 reverse	CCGGATTTTGGCGTATCAGAC
Wnt5a forward	TCGACTATGGCTACCGCTTTG
Wnt5a reverse	CACTCTCGTAGGAGCCCTTG
Wnt6 forward	GGCAGCCCCTTGGTTATGG
Wnt6 reverse	CTCAGCCTGGCACAACTCG
Wnt10a forward	GGTCAGCACCCAATGACATTC
Wnt10a reverse	TGGATGGCGATCTGGATGC
Wnt10b forward	GTGAGCGAGACCCCACTATG
Wnt10b reverse	CACTCTGTAACCTTGCACTCATC
PTH1R forward	CTGGGCATGATTTACACCGTG
PTH1R reverse	CAGTGCAGCCGCCTAAAGTA
